# Secondary Prevention in Lower Extremity Artery Disease Patients: Lipid-Lowering Therapy and Long-Term Guideline Adherence

**DOI:** 10.3390/jcm11226838

**Published:** 2022-11-19

**Authors:** Linda Mueller, Christiane Engelbertz, Holger Reinecke, Eva Freisinger, Nasser M. Malyar, Matthias Meyborg, Tobias J. Brix, Julian Varghese, Katrin Gebauer

**Affiliations:** 1 Department of Cardiology I—Coronary and Peripheral Vascular Disease, Heart Failure, University Hospital Muenster, 48149 Muenster, Germany; 2 Institute of Medical Informatics, University of Muenster, 48149 Muenster, Germany

**Keywords:** lower extremity artery disease, lipid-lowering therapy, secondary prevention

## Abstract

Lower extremity artery disease (LEAD) affects millions of elderly patients and is associated with elevated cardiovascular morbidity and mortality. Risk factor modification, including the therapy of dyslipidaemia, is mandatory to reduce cardiovascular event rates and to improve survival rates. However, only a minority achieve the recommended low-density lipoprotein cholesterol (LDL-C) target level < 55 mg/dL, according to the current ESC/EAS guidelines on the treatment of dyslipidaemia. This study elucidated the implementation of the lipid-lowering guideline recommendations of 400 LEAD patients with LDL-C > 100 mg/dL and their adherence to treatment adjustment during follow-up. Despite a sustained statin prescription in 93% of the patients, including 77% with high-intensity statins at follow-up, only 18% achieved the target level. Ezetimibe appeared in 21% and LDL-C goals were reached significantly more often with combination therapy. Recurrent revascularization appeared more often (28%) than coronary artery or cerebrovascular disease progression (14%) and 7% died. Despite the frequent use of high-intensity statins and expandable rates of ezetimibe, the progression of cardiovascular events remained inevitable. Only 18% of the patients had received recommendations on lifestyle modification, including dietary adaptations, which is key for a holistic approach to risk factor control. Thus, efforts for both pharmacological and behavioral strategies are needed to improve clinical outcomes and survival rates.

## 1. Introduction

Lower extremity artery disease (LEAD) is a highly prevalent disease in the elderly patient population. In Germany, 2.3 million patients were affected by LEAD in 2018 [1]. According to the ambulatory claims data of statutorily insured patients in Germany, the frequency of PAD diagnoses increased between 2009 and 2018, from 1.85% to 3.14% [1].

Secondary prevention, including lipid-lowering therapy (LLT), is mandatory in this high-risk patient population. Reducing risk factors by lifestyle modification (e.g., diet, smoking cessation and physical activity), pharmacotherapy (LLT, antiplatelet and blood pressure medication) and optimal adjustment of diabetes mellitus are further essential components of guideline-adherent treatment.

The 2019 guidelines of the European Society of Cardiology (ESC) and the European Atherosclerosis Society (EAS) for the management of dyslipidaemias in LEAD patients recommend a maximum acceptable dose of a high-intensity statin (atorvastatin ≥ 40 mg or rosuvastatin ≥ 20 mg), accompanied by ezetimibe or a proprotein convertase subtilisin/kexin type 9 (PCSK9) inhibitor, if necessary, in order to diminish the risk of atherosclerotic cardiovascular disease events [2]. According to these ESC/EAS guidelines, a decline in low-density lipoprotein cholesterol (LDL-C) of greater than 50% and a target LDL-C-level of <55 mg/dL in patients with atherosclerotic cardiovascular disease (ASCVD) is recommended [2]. A reduction of 39 mg/dL LDL-C (1 mmol/L) translates into a cardiovascular risk reduction of 20%. However, in a recent analysis of LLT use in a subset of LEAD patients, LDL-C target level achievement was present in only 19% of patients [3].

The guideline-recommended medication of statins in a cohort of 2.3 million LEAD patients in Germany increased from 43% in 2009 to 56% in 2016, which may be due to an increased awareness of secondary prevention treatment; however, this still leaves the need for improvement regarding guideline adherence [1]. In another large chronic LEAD cohort (*n* = 241,375), the prescription of LLT agents was present in only 30% of the cases [4]. LEAD that is mostly localized in the lower extremities causes an impairment in patients’ quality of life, e.g., by reducing patients´ walking ability [5]. Even asymptomatic LEAD patients have greater functional impairment and faster functional decline than those without LEAD, potentially reflecting the burden of unbalanced risk factors [6].

Coronary artery disease (CAD) and cerebrovascular disease (CVD) share the same cardiovascular risk factors, of which smoking and diabetes mellitus are the most important ones [7]. An association has been found between LEAD and an enlarged risk of cardiovascular morbidity and mortality [7]. Among all patients with atherosclerotic disease, LEAD has the worst cardiovascular morbidity and mortality rates [8]. Hence, strict adherence to guideline-recommended secondary prevention leads to an improvement in this deleterious version of atherosclerosis. The reasons for non-adherence to secondary prevention are manifold. A failure to start LLT or to administer dose adjustments to avoid potential side effects, a reluctance to choose statins with higher potential, the need for combination therapy, and even the discontinuation of LLT have been previously revealed causes of insufficient guideline adherence. An unawareness of the applicable guidelines with LDL-C target levels may account for suboptimal adherence and missing incentives for target level achievements in the reimbursement of preventive measures may be additive [3,4,9,10,11]. So far, the data about LLT adjustments and written recommendations in the doctors’ reports during hospital treatment are missing, elucidating one of the reasons for the suboptimal guideline compliance. In this study, LLT treatment and LDL-C target level achievement in LEAD patients at a tertiary care center were evaluated with regard to their potential for improvement.

## 2. Methods

### 2.1. Data Source and Study Population

A retrospective investigation of hospital patient records was conducted by analyzing LEAD patients in the period from January 2017 to February 2020, who were treated as in- or outpatients in the Department of Cardiology I—Coronary and Peripheral Vascular Disease, Heart Failure of the University Hospital Muenster. Patients were selected according to ICD (International Statistical Classification of Diseases and Related Health Problems) codes (I70.2*, atherosclerosis of native arteries of the extremities) of the medical information system. Thus, 400 LEAD patients were included. Patients with rest pain, lower limb ulceration >2 weeks or gangrene, indicated by ICD codes I70.23, I70.24 and I70.25, respectively, were categorized as patients with chronic limb-threatening ischemia (CLTI).

Additional inclusion criteria applied as follows: LDL-C >100 mg/dL and patients ≥18 years of age.

Each LEAD patient was enrolled in a structured follow-up (FU) program at our facility, resulting in yearly encounters, or more frequently in case of clinical urgency. Follow-up information was extracted between March 2020 and June 2021 from the hospital’s patient records to allow each subject to participate at least in one FU visit after the period of data acquisition (January 2017 to February 2020). Due to the COVID-19 pandemic, FU visits were postponed for some subjects, so the period of FU data collection was extended to 16 months.

Again, data on the use of LLT were collected, as well as LDL-C-values, if available. Furthermore, information on recurrent endovascular revascularization, progression of other atherosclerotic diseases and survival was collected.

Data were anonymized for statistical analysis. The trial was approved by the Ethics Committee of the University of Muenster (reference number 2021-221-f-S).

### 2.2. Patient Data Collection

Blood parameters—including hemoglobin (g/dL), leukocytes (thousand/µL), thrombocytes (thousand/µL), thyroid-stimulating hormone (TSH) (µU/L), serum creatinine (mg/dL), estimated glomerular filtration rate (eGFR; mL/min/1.73 m^2^), total cholesterol (TC; mg/dL), high-density lipoprotein cholesterol (HDL-C; mg/dL), directly measured LDL-C (mg/dL) and triglycerides (mg/dL)—were extracted from the patient records. LLT at admission and discharge, height, weight, risk factors for cardiovascular disease (hypertension, smoking, diabetes mellitus, family disposition of cardiovascular disease) and treatment recommendation regarding secondary prevention were extracted from the doctors’ reports. Treatment recommendation included dietary advice and information on LLT in the epicrisis.

For LLT, the following drugs were documented: statins (atorvastatin, simvastatin, rosuvastatin, pravastatin, fluvastatin, lovastatin); ezetimibe; PCSK9 inhibitors and other LLT (fibrates and bile acid sequestrants). Daily doses of statins at admission or at ambulatory presentation at the angiological outpatient department and its changes at discharge were documented, as well as the use of ezetimibe. Atorvastatin and rosuvastatin were considered high-intensity statins, as these are known to be highly efficient in reducing LDL-C by more than 50% with moderate to high doses (atorvastatin ≥ 40 mg or rosuvastatin ≥ 20 mg) and are considered first line substances for LDL-C lowering. Statin-naive patients were those without previous statin medication. To elucidate changes in statin application differences at admission compared to discharge or changes at ambulatory visits were calculated as follows: number of statins at discharge divided by number of statins at admission, then multiplied by 100. The results were subtracted from 100 and displayed as percentage related to initial statin rates. To elucidate differences in prescription patterns before and after introduction of the 2019 ESC/EAS guidelines on the management of dyslipidaemias, statin and ezetimibe use, as well as LDL-C values, were analyzed with respect to the inclusion date.

Patients with LDL-C < 55 mg/dL at FU were considered target level achievers, as the current ESC/EAS guidelines on the treatment of dyslipidaemias recommends an LDL-C value lower than 55 mg/dL in LEAD patients. Patients exceeding an LDL-C level of 55 mg/dL at FU were categorized as non-target level achievers, correspondingly.

Anticoagulant and antiplatelet therapy were counted at the time of admission or at presentation at the outpatient department of our facility. As anticoagulants, phenprocoumon, apixaban, rivaroxaban, edoxaban and dabigatran were analyzed. The antiplatelet therapies, acetylsalicylic acid (ASA) and clopidogrel, or a combination of both were counted.

### 2.3. Statistical Analysis

Data were analyzed with IBM© SPSS© Statistics Version 28.0.1.0 (IBM, Armonk, NY, USA). Distribution of comorbidities and LLT use are shown in absolute numbers and percentages in brackets. Laboratory values are displayed as mean values with standard deviation (SD). Differences in categorical variables between the groups of target level achievers and non-achievers concerning the LLT, as well as the distribution of statin medication at admission and discharge, and at discharge and follow-up, respectively, were analyzed by Chi-square test (*p* < 0.05 was considered statistically significant). Differences in continuous variables, such as LDL-C values at admission and follow-up, were analyzed by *t*-test (*p* < 0.05 was considered statistically significant).

#### Aims and Outcomes

The rationale of this work was to elucidate the change in LLT and explicit references of LLT and dietary modification in the epicrisis of doctors’ reports in a high-risk patient population, extensively exceeding LDL-C target levels. LLT and its effect on LDL-C target level achievement, as well as clinical outcomes and survival rates, have been studied to reveal potential for improvement in secondary prevention in this high-risk patient population.

## 3. Results

In this study cohort of 400 patients being treated for LEAD, the average age was 68 (SD 11) years and 63% were male. The proportion of CLTI patients was 30%, the remaining 70% were categorized as claudicants. The average body mass index (BMI) was 26.7 kg/m^2^ (SD 4.8 kg/m^2^) and 22% were obese (BMI > 30 kg/m^2^). In total, 82 (20%) were treated as outpatients and 318 (80%) as inpatients. Baseline characteristics, including comorbidities, laboratory parameters, background anticoagulation and platelet inhibitor medication, are shown in Table 1.

At admission, 38% of all the patients did not take any statins (Table 2). Both simvastatin (27%) and atorvastatin (28%) were recorded in similar proportions. Rosuvastatin was present in 3%, thus, high-intensity statin use accounted for 31%. The changes in statin applications between admission and discharge were statistically significant (*p* < 0.001).

Moreover, at discharge, 21% of the patients were taking ezetimibe, only three patients (1%) received PCSK9 inhibitors and none of the patients received other LLTs (fibrates and bile acid sequestrants). One patient passed away during their hospital stay and was expelled from further analysis.

**Figure 1:**
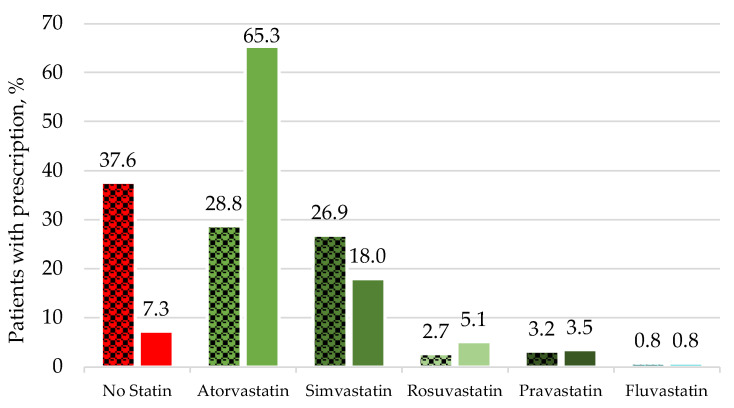
Changes of statin application during hospital treatment. The distribution of statin application is displayed for admission or at ambulatory contact at the angiolocigal outpatient department (dotted bars) and discharge, or after ambulatory presentation at the angiological outpatient department (solid bars), in %. Columns in red display no statin treatment, columns in green display statin treatment (green: atorvastatin, dark green: simvastatin, light green: rosuvastatin, olive: pravastatin, greenish-blue: fluvastatin).

At discharge, 7% still had no statins, whereas 65% were on atorvastatin and 18% were on simvastatin, respectively. Rosuvastatin was prescribed in 5% of the cases, so the proportion of high-intensity statins, including atorvastatin and rosuvastatin, more than doubled: from 31% at admission to 70% at discharge (Figure 1).

Regarding the 7% of patients without statins at discharge, none of them had statin medication at admission either and, therefore, remained statin-naïve during their medical contact. The distribution of further statin formulations are displayed in Figure 1.

### 3.1. Change in Statin Classes during Hospital Treatment

**Figure 2:**
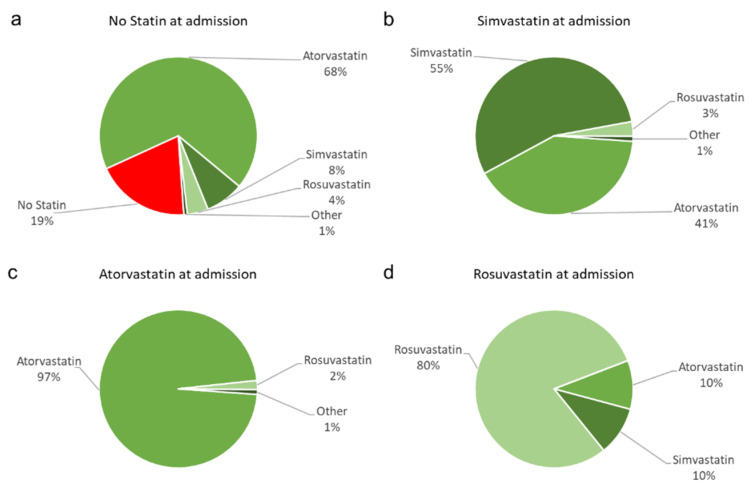
Changes in statin agents, according to initial treatment at admission. (**a**) For patients with no statin prescription at admission, the prescription practices at hospital discharge or after presentation as outpatient in the angiological department of our facility with different statins are displayed in %. Of note, 19% received no statin prescription at discharge (red slice). For patients with (**b**) simvastatin, (**c**) atorvastatin and (**d**) rosuvastatin at admission, the prescription practices at hospital discharge or after presentation as outpatient in the angiological department with different statins are displayed in %. Red color display no statin treatment, green colors display statin treatment (green: atorvastatin, dark green: simvastatin, light green: rosuvastatin, olive: other.

Of those without statins at admission, 68% received atorvastatin and received 4% rosuvastatin, respectively, when leaving the hospital, whereas 9% were prescribed statins of a lower intensity. Another 19% remained statin-naïve (Figure 2a). Patients on simvastatin at admission mainly stayed on this statin type (55%, Figure 2b). Those primarily on high-intensity statins (atorvastatin and rosuvastatin) predominantly remained on this statin type or switched to another high-intensity statin (Figure 2c,d).

### 3.2. Unused Potential of LLT Enhancement during Hospital Treatment

Of the 232 patients with statins at admission or at presentation at the angiological outpatient department and, by the definition of this study population, exceeding LDL-C target level achievement (LDL-C >100 mg/dL), 30% remained with a statin of low- or intermediate-intensity.

Of all the patients on atorvastatin or rosuvastatin at discharge, 48% or 13%, respectively, had no dose titration despite exceeding the LDL-C target level and, therefore, did not experience the guideline-recommended dose adjustment of their statin medication. Moreover, the proportion of ezetimibe use in the atorvastatin-treated patients was 36%, and in the rosuvastatin-treated patients was 38%; however, in patients with simvastatin, more than two thirds (70%) had no dose escalation of their statin medication and only 16% had concomitant ezetimibe medication.

Two patients were switched from a high-intensity statin to one of a lower intensity. Ezetimibe use, in general was present in only 21% of cases. The proportion of PCSK9 inhibitor application was scarce, at only 1% of cases (*n* = 3).

### 3.3. Recommendations on Lifestyle, Dietary and Pharmacological Changes in the Doctors’ Reports

**Figure 3:**
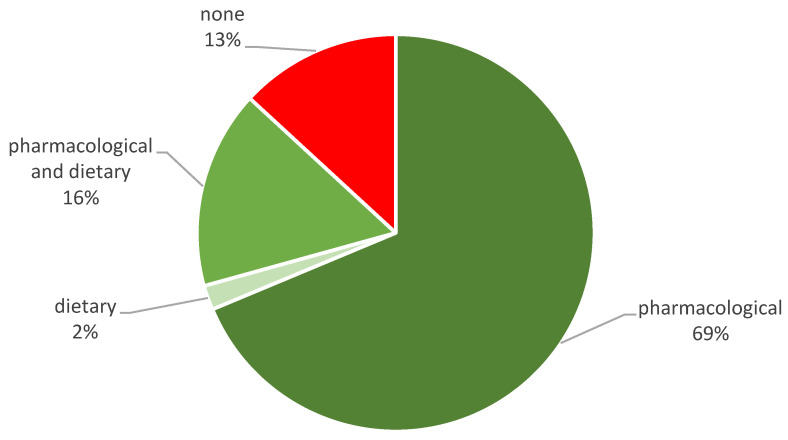
Recommendation of lipid-lowering therapy in the epicrisis of the doctors’ reports. Four different recommendation options are displayed: pharmacological advice (dark green), dietary advice (light green), both pharmacological and dietary advice (green), no advice (red). Values are shown in %.

In the epicrisis of the doctors’ reports, 68% received the medication recommendation, 2% received dietary advice, 16% received both medication and dietary advice, and 13% did not receive any of these (Figure 3). One patient (0.3%) discontinued LLT.

### 3.4. Follow-Up

The follow-up data of 327 patients, with a median of 564 days, were collected and evaluated, showing that 6% were still not receiving any statins, but that 77% received a high-intensity statin (66% atorvastatin and 11% rosuvastatin), in contrast to the 74% at discharge (Table 3). The percentage of patients receiving ezetimibe increased to 28%, from 23% at discharge after their initial presentation. Five patients (2%) were prescribed PCSK9 inhibitors.

Overall, recurrent revascularization was necessary in 28% of the patients, and the progression of CAD or CVD occurred in 14% of the patients and 29 subjects died (7%).

#### 3.4.1. L-C Target Level Achievement with Respect to LLT and Outcome Parameters

LDL-C values were retrieved for 168 patients at follow-up (Table 4). Only 18% of them had an LDL-C < 55 mg/dL and were, thus, achieving LDL-C target levels, according to the guideline recommendations. The average LDL-C at follow-up was 78 mg/dL (SD 29) and, therefore, declined significantly from the admission values (132 mg/dL; SD 28, *p* < 0.001).

Patients with target level achievement had more revascularizations (68%) than those with LDL-C >55 mg/dL (48%; *p* = 0.07). The progression of CAD and CVD appeared in 23% of the patients, with LDL-C <55 mg/dL, compared to 19% in those with LDL-C above target level (non-significant (n.s.)).

High-intensity statin use was present in 89% of those with LDL-C <55 mg/dL, whereas 85% of the patients above the LDL-C target level had high-intensity statin medication (n.s.).

The average dose of statins for patients with LDL-C <55 mg/dL was 44 mg per day (SD 21) in atorvastatin; 38 mg per day (SD 20) in simvastatin users; and 24 mg per day (SD nine) in rosuvastatin, respectively. Patients with ezetimibe as an add-on to statin medication reached LDL-C target levels significantly more frequently (59% vs. 35%, *p* = 0.02). PCSK9 inhibitor use was documented in 7% of patients below LDL-C target levels, in contrast to 2% in those above target level (n.s.).

Two patients with LDL-C < 55 mg/dL died, compared to eight in the LDL-C > 55 mg/dL group (n.s.).

#### 3.4.2. Differences in Prescription Patterns before and after Introduction of the 2019 ESC/EAS Guidelines on the Management of Dyslipidaemias

The proportion of high-intensity statin prescriptions (atorvastatin and rosuvastatin) at discharge and before and after the release of the 2019 ESC/EAS guidelines on the management of dyslipidaemias was 70% and 78%, respectively. The proportion of simvastatin use before and after the 2019 guidelines was 19% and 11%, respectively. The differences in statin prescriptions were significant with respect to the time of inclusion (before or after the 2019 guideline release). The prescription of ezetimibe at discharge was present in 20% of patients before and 27% of patients after the 2019 dyslipidaemia guidelines and was even more pronounced at FU (27% vs. 36%, *p* = n.s.). This indicates a shift towards a more intense high-intensity statin and ezetimibe use following the release of the guideline recommendations.

However, this did not translate into differences in LDL-C changes. At admission, the mean LDL-C values before the 2019 dyslipidaemia guidelines were 131 ± 28 mg/dL, in contrast to 141 mg/dL ± 23 mg/dL (*p* = n.s.) after the 2019 dyslipidaemia guidelines. At follow-up, which was performed between March 2020 and June 2021, the mean LDL-C value was 77 ± 29 mg/dL in the patients included before the 2019 guidelines, and 82 ± 29 mg/dL (*p* = n.s.) in those included after the guideline release, respectively. Overall, the percentage change in LDL-C lowering was 42%, irrespective of the patient’s inclusion date. The numerically higher mean LDL-C values at FU in patients included after the 2019 guideline implementation could be explained by more elevated baseline LDL-C values. (Supplementary Table S1).

## 4. Discussion

This retrospective study was conducted to elucidate guideline adherence in the secondary prevention of LEAD patients. Data on guideline adherence regarding LLT in LEAD patients are scarce. Moreover, precedent analyses proved the frequency of LLT in this ASCVD patient population to be insufficient, however, the reasons for this undertreatment remain unknown [3].

Therefore, this study has added valuable information to the approach of optimizing LLT in LEAD patients with an LDL-C level profoundly out of target level (>100 mg/dL) and to its durability over time—with respect to guideline-adherent adjustments to LLT, LDL-C target level achievement, and its effect on clinical outcome parameters (recurrent revascularization, progression of CAD and CVD, and death).

In total, 67% of the subjects had either no statin medication or received non-guideline recommended statins of low or intermediate potential at admission. The efforts to improve secondary prevention resulted in a rise in high-intensity statin recommendations to 70%, which even reached 77% at follow-up. Nonetheless, target level achievement with LDL-C < 55 mg/dL at follow-up was present in only 18% of the patients. These results were in line with other large retrospective data analyses for ASCVD patients who were treated suboptimally [13].

A large population-based analysis of LEAD patients (*n* = 194,151) revealed a statin prescription rate of 79.0%, however, the proportion of guideline-concordant statin intensity was 40.9% [14]. This observation was recently confirmed by another single center study, with 956 LEAD patients whose LDL target level achievement was 19%, and a prescription rate of high-intensity statins of only 41% [15]. Moreover, great inter-facility differences concerning the maintenance of statin prescriptions, irrespective of patient-inherent factors, could be elucidated. This suggests that a LEAD patient should receive a guideline-adherent LLT, including statin intensity adaptation, before discharge or after ambulatory presentation at the angiological outpatient department of a tertiary care center, in order to sufficiently maintain secondary prevention medication in the long term [14].

Firstly, dose titration of existing statin medications, as recommended by the current guidelines on secondary prevention, is one cornerstone of LDL-C target level achievement but was ignored by the majority of the patients, despite an LDL-C level >100 mg/dL [2]. This is a major tool for improving target level achievement and should be implemented regularly. The reasons for therapeutic inertia are manifold. The avoidance of the potential side effects of higher statin doses, following the fire and forget strategy instead of the treat to target approach, are among the possible explanations for sticking to the insufficient statin regime once established. A lack of incentives for guideline adherence in the secondary prevention of ASCVD patients may contribute to the vast majority who are out of the LDL-C target level.

Secondly, the use of ezetimibe showed significantly improved target level achievement (59% vs. 35%, *p* = 0.02). The proportion of patients with a cholesterol absorption inhibitor was still low at follow-up (28%), ignoring another LDL-C lowering potential of 20–30% in addition to the recommended >50% LDL-C reduction by high-intensity statins. In patients who were at high-risk of subsequent major adverse cardiac events (MACE) after coronary syndrome, ezetimibe was efficient in reducing recurrent myocardial infarction and stroke [16]. Considering the average LDL-C-level of 78 mg/dL at follow-up, the addition of ezetimibe could account for the LDL-C target level achievement in the majority of the patients. The application of a statin with ezetimibe as a fixed-dose combination was even more beneficial than a separate pill combination as far as LDL-C lowering was concerned and could, therefore, be a promising strategy for more profound target level achievement [17]. Furthermore, the application of bempedoic acid, which was not available during data acquisition, in combination with ezetimibe may be promising in producing a profound reduction in LDL-C, irrespective of background statin medication [18], even though the LDL-C lowering effect of both statins and ezetimibe, as well as bempedoic acid, is of great inter-individual variability [19,20]. It is, therefore, of great importance to switch patients to high-intensity statins and to titrate statins at the maximum tolerable dose, and it is even more important to add other oral LLTs—such as ezetimibe and, potentially, bempedoic acid—before an evaluation of therapeutic escalation with PCSK9 inhibitors. With a diligent selection of LLT, a customized approach of LDL-C-lowering in order to achieve target levels is mandatory and most likely to be successful if combination therapy is applied and sustained.

### 4.1. Treatment Recommendation for Dietary Adaptations

In the epicrisis of the doctors’ reports, explicitly mentioned treatment recommendations for LLT were present in 68% of the reports, whereas the necessity of dietary adaptations could be captured in only 18% of them. Lifestyle modifications and dietary counselling are two of the cornerstones of sufficient secondary prevention in this high-risk patient population. Whereas LLT is frequently prescribed, directly addressing the potential of nutritional adaptations with an LDL-C lowering effect should be intensified. The combination of medical treatment with powerful lipid-lowering agents and behavioral changes in lifestyle and nutrition must be communicated by medical staff and should be sustained by participation in rehabilitation programs. This therapeutic strategy was inefficiently used in a longitudinal observational study of LEAD patients, in which only 22% of the patients had dietary counselling [21]. Therefore, continuing education and counselling exceeding the immediate intervention is necessary to ensure sustained guideline-adherent secondary prevention. Patients’ and physicians’ awareness of the achieved risk factor control and of the remaining potential for improvement is key to achieving the guideline goals. The implementation of disease management programs as well as digital tools—e.g., secondary prevention apps linked with reimbursement benefits in health insurance—may be supportive.

Finally, a comprehensive approach to the secondary prevention of LEAD, apart from sole pharmacological treatment, is necessary. Behavioral strategies and supervised exercise programs have been proven to reduce morbidity and mortality and are cost-effective, however, they are infrequently prescribed [22]. Structured programs for lifestyle and risk factor modification are beneficial in achieving treatment goals [23].

Additionally, providing advice about nutrition adaptation being efficacious on lipid-lowering should be a standard approach, however, a minority of patients are presented with this preventive strategy [24].

All these efforts should be made for every single patient, based on individualized secondary prevention, to utilize all available measures and accomplish the urgent need of sufficient risk factor control.

### 4.2. Effects on Repeat Revascularization

Repeat revascularization is one component of major adverse limb events (MALE), which could be numerically reduced by achieving very low LDL-values in PCSK9 inhibition by evolocumab [25]. LEAD patients using statins are at a profoundly lower risk of MACE, however, the impact of different LDL-C target levels on MALE remains unknown, as randomized trials in LEAD patients answering this urgent clinical question have not been undertaken yet [26,27]. A recent meta-analysis of different LLT regimes in LEAD patients, including statin intensities, revealed a reduction in all-cause mortality, but no improvement in MALE with high-intensity statins application [28].

In our cohort, the number of recurrent peripheral revascularization was increased in the group with LDL-C target level achievement. This effect may be explained by more frequent contact with their doctors and a more compliant patient behavior, which leads to increased examination and treatment necessity. Another explanation may be that LEAD is a very malignant version of ASCVD. The rates of progression of CAD and CVD were frequent, irrespective of LDL-C target level achievement. The elevated rate of myocardial infarction and stroke in LEAD patients is profoundly responsible for their cardiovascular mortality excess rate, compared to other ASCVD phenotypes. The intense endovascular treatment of LEAD, despite LDL-C levels <55 mg/dL, may, thus, be irrespective of cardiovascular outcomes. The death rate of 7% and 6% in target level achievers and non-achievers supports this assumption. Effects of profound and sustained LDL-C lowering on cardiovascular survival and MALE may be seen in long-term follow-up only, as it has been shown before [29].

### 4.3. Cardiovascular Outcome

Intensified LDL-C lowering in the cardiovascular outcomes in LEAD patients was beneficial [30]. LDL-C lowering by 39 mg/dL (1 mmol/L), with intensive statin use, translated into a risk reduction of 20% in a patient population, with or without established ASCVD. It is noteworthy that more intensive LDL-C lowering leads to better outcome concerning major cardiovascular events [31].

In this study, the rate of progression of CAD and CVD was 14% during follow-up. LEAD patients are at extraordinarily increased risk of cardiovascular events, which are the main reasons for the excess mortality compared to other ASCVD patients [29]. Statin-driven LDL-C lowering results in plaque stabilization and can be accounted for as mechanisms of cardiovascular event reduction [32]. These observations have been confirmed in PCSK9 inhibitor trials, in which very low LDL-C values resulted in plaque modification [33,34]. However, in LEAD patients with advanced atherosclerosis, sometimes present in more than one vascular territory, sustained and profound LDL-C lowering may be needed until treatment translates into clinical effect.

## 5. Limitations

First, this study comprised data from a single tertiary care center. Follow-up information was present for only 82% of the patients and the LDL-C values for the calculation of target level achievement was even less frequently documented (52%). A selection bias can, therefore, not be excluded.

Second, subsequent treatment could potentially have taken place with the primary care physician or family doctor. Hence, the information on survival or treatment adaptation could have been incomplete. However, all LEAD patients are encouraged to attend at least yearly follow-up visits at our facility so that data acquisition is thorough in those recurring.

Third, with underlying ASCVD comorbidities, the reason for LLT could have been triggered by diseases other than LEAD and, therefore, it has to be acknowledged as a potential confounder. Additionally, other medications frequently applied in ASCVD, such as anticoagulants and antiplatelet therapeutics, may have contributed to the outcome parameters.

Fourth, written recommendations for lifestyle counselling or medication plans might have been discarded by the patients and/or physicians, which may have influenced LDL-C target level achievement.

Fifth, the “*2019 ESC/EAS* *Guidelines for the management of dyslipidaemias: Lipid modification to reduce cardiovascular risk*” came into effect in August 2019, whereas the period of retrospective data collection commenced in January 2017. However, follow-up was performed between May 2020 and June 2021, where the recent guidelines had to be applied. Moreover, the former 2016 ESC/EAS guidelines for the management of dyslipidaemias already demanded a reduction in LDL-C ≥ 50% from baseline and statin dose titration, if target level achievement, which was set to <70 mg/dL LDL-C, was missing [35]. Therefore, the treatment algorithm did not differ from the one used in the current version.

Nevertheless, this study comprised 400 consecutive LEAD patients, who had been referred to a tertiary care center for dedicated treatment by primary care physicians, general practitioners, cardiologists, angiologist and vascular surgeons and, thus, represented an unselected patient population. This work elucidated, in real life, the implementation of secondary prevention strategies for LLT in LEAD patients and showed the reasons for the non-achievement of guideline goals.

## 6. Conclusions

The target level achievement of LDL-C (<55 mg/dL) can be significantly improved by adding ezetimibe on top of statin medication, which should be applied to the large proportion of non-target level achievers. A guideline-concordant switch from statins of low- or intermediate-intensity to those of high-intensity and dose titration may be beneficial in the reduction in cardiovascular morbidity and mortality. Advice on behavioral changes, including dietary adaptations and exercise efforts, in an approach of shared decision making, in addition to prescribed medication, plays a key role for secondary prevention to the full extent.

## Figures and Tables

**Table 1:** Baseline characteristics of the study cohort.

Characteristics	Patients *n* = 400 (100%)
Men, *n* (%)	252 (63)
Age, year (SD)	68 (11)
Inpatients, *n* (%)	318 (80)
CLTI, *n* (%)	119 (30)
**Comorbidities**	
Hypertension, *n* (%)	326 (82)
Nicotine use, former, *n* (%)	137 (34)
Nicotine use, ongoing, *n* (%)	164 (41)
Diabetes mellitus, *n* (%)	115 (29)
Family history of CAD, *n* (%)	106 (27)
Chronic kidney disease *	
G1 (>90 mL/min), *n* (%)	102 (28)
G2 (60–89 mL/min), *n* (%)	140 (38)
G3a (45–59 mL/min), *n* (%)	59 (16)
G3b (30–44 mL/min), *n* (%)	25 (7)
G4 (15–29 mL/min), *n* (%)	21 (6)
G5 (<15 mL/min), *n* (%)	18 (5)
BMI, kg/m^2^ (SD)	26.7 (4.8)
BMI >30 kg/m^2^, *n* (%)	84 (22)
Number of secondary diagnoses ^#^, *n* (SD)	8 (5)
**Laboratory values**	
Total cholesterol, mg/dL, median (min;max)	196 (145;388)
average (SD)	204 (35)
LDL-Cholesterol, mg/dL, median (min;max)	123 (100; 233)
average (SD)	132 (28)
HDL-Cholesterol, mg/dL, median (min;max)	50 (18;143)
Average (SD)	53 (17)
Triglycerides, mg/dL, median (min;max)	157 (47; 995)
average (SD)	154 (33)
Hemoglobin, g/dL, median (min;max)	13.5 (7.7; 18.0)
average (SD)	13.4 (1.9)
Creatinine *, mg/dL, median (min;max)	1.0 (0.4; 11.4)
average (SD)	1.3 (1.2)
eGFR*, mL/min/1.73 m^2^, median (min;max)	73 (4; 117)
average (SD)	69 (26)
Thrombocytes, thousand/µL, median (min;max)	253 (75; 629)
average (SD)	268 (81)
Leukocytes, thousand/µL, median (min;max)	8.2 (2.8; 18.2)
average (SD)	8.4 (2.5)
TSH, µU/L, median (min;max)	1.7 (0.1;18.7)
average (SD) (SD)	2.0 (1.7)
**Anticoagulants**	
Phenprocoumon, *n* (%)	43 (11)
Apixaban, *n* (%)	26 (7)
Rivaroxaban, *n* (%)	12 (3)
Edoxaban, *n* (%)	6 (2)
Dabigatran, *n* (%)	4 (1)
**Platelet inhibitors**	
ASA, *n* (%)	131 (33)
Clopidogrel, *n* (%)	106 (27)
ASA and Clopidogrel, *n* (%)	42 (11)

^#^—inpatients only (*n* = 318); *—data available in *n* = 365; ASA—acetylsalicylic acid; CAD—coronary artery disease; CLTI—chronic limb-threatening ischemia; eGFR—estimated glomerular filtration rate; HDL—high-density lipoprotein; LDL—low-density lipoprotein; SD—standard deviation; min.—minimum; max.—maximum. Calculation of kidney impairment by eGFR according to KDIGO classification [12].

**Table 2:** Statin medication at admission and discharge.

Statin Medication	At Admission	At Discharge	%Change from Admission
No statin, *n* (%)	140 (38)	27 (7)	−81
Atorvastatin, *n* (%)	107 (28)	243 (65)	+127
Simvastatin, *n* (%)	101 (27)	67 (18)	−33
Rosuvastatin, *n* (%)	10 (3)	19 (5)	+90
Pravastatin, *n* (%)	12 (3)	13 (3)	+8
Fluvastatin, *n* (%)	3 (1)	3 (1)	0
Ezetimibe ^+^, *n* (%)	n/a	85 (21)	n/a
PCSK9 inhibitor ^+^, *n* (%)	n/a	3 (1)	n/a

Admission data on statin therapy available for 373 patients. Changes between admission and discharge statistically significant (*p* < 0.001). ^+^—only data at discharge available. n/a—not applicable. PSCK9—proprotein convertase subtilisin/kexin type 9.

**Table 3:** Follow-up data on lipid-lowering therapy and clinical outcomes.

	Admission	Discharge	Follow-Up
**Lipid-lowering therapy, *n* = 327**			
No statin, *n* (%)	110 (36)	17 (5)	21 (6)
Atorvastatin, *n* (%)	90 (30)	221 (68)	217 (66)
Rosuvastatin, *n* (%)	9 (3)	18 (6)	36 (11)
Simvastatin, *n* (%)	80 (27)	58 (18)	43 (13)
Other low-intensity statins, *n* (%)	13 (4)	13 (4)	10 (3)
PSCK9 inhibitor, *n* (%)	n/a	3 (1)	5 (2)
Additional ezetimibe, *n* (%)	n/a	74 (23)	91 (28)
**Clinical outcome parameter, *n* = 327**			
Recurrent revascularization, *n* (%)			93 (28)
Progression of CAD/CVD, *n* (%)			44 (14)
**Survival**			
Death, *n* (%)			23 (7)
Death in CLTI patients, *n* (%)			11 (11)
Death in claudicants, *n* (%)			12 (5)

CAD—coronary artery disease; CVD—cerebrovascular disease.

**Table 4:** Follow-up data on LDL-C target level achievement.

Laboratory Values at Follow-Up, *n* = 168	LDL-C < 55 mg/dL	LDL-C ≥ 55 mg/dL	*p*-Value
Patients, *n* (%)	31 (18)	137 (82)	n.s.
No statin treatment, *n* (%)	0 (0)	6 (4)	n.s.
Treatment with high-intensity statin, *n* (%)	26 (84)	116 (85)	n.s.
Treatment with additional ezetimibe ^#^, *n* (%)	16 (59)	48 (35)	0.02
Treatment with PCSK9 inhibitor, *n* (%)	2 (7)	3 (2)	n.s.
Recurrent revascularization, *n* (%)	21 (68)	66 (48)	n.s.
Progression of CAD/CVD, *n* (%)	7 (23)	26 (19)	n.s.
Death, *n* (%)	2 (7)	8 (6)	n.s.

^#^ Data available for *n* = 163. CAD—coronary artery disease; CVD—cerebrovascular disease; n.s.—non-significant.

## Data Availability

Data are available upon request from K.G.

## Supplementary Materials

The following supporting information can be downloaded at: https://www.mdpi.com/article/10.3390/jcm11226838/s1. Table S1: Distribution of statin prescription and LDL-C values related to inclusion date.
