# Supplementary material for: Secondary Prevention in Lower Extremity Artery Disease Patients: Lipid-Lowering Therapy and Long-Term Guideline Adherence
Source: J Clin Med. 2022 Nov 19;11(22):6838. doi: 10.3390/jcm11226838 (PMC9692475; doi:10.3390/jcm11226838)
Supplement: Supplementary file 1 [file jcm-11-06838-s001.zip › jcm-1974520-supplementary.pdf]

Supplements to

Secondary Prevention in Lower Extremity Artery Disease Patients: Lipid-Lowering Therapy and Long-Term Guideline Adherence

Mueller L et al.

Supplementary Table S1. Distribution of statin prescription and LDL-C values related to inclusion date

	Admission	Discharge	Follow-up
Atorvastatin, %			
Pre 2019 guideline	28	67	66
Post 2019 guideline	34	62	68
Rosuvastatin, %			
Pre 2019 guideline	1	3	10
Post 2019 guideline	11	16	16
High-Intensity Statin, %			
Pre 2019 guideline	29	70	76
Post 2019 guideline	43	78	84
Simvastatin, %			
Pre 2019 guideline	29	19	14
Post 2019 guideline	17	11	7
p-value	<0.001	<0.004	n.s.
Ezetimibe, %			
Pre 2019 guideline	n/a	20	27
Post 2019 guideline	n/a	27	36
p-value	n/a	n.s.	n.s.
LDL-C, mg/dl; mean ± SD			
Pre 2019 guideline	131±28	n/a	77±29
Post 2019 guideline	141±23	n/a	82±29
p-value	n.s.	n.s.	
